# All parts of the WHO *Mycobacterium tuberculosis* mutation catalog need to be applied when evaluating its performance

**DOI:** 10.1128/spectrum.02157-24

**Published:** 2025-04-02

**Authors:** Sacha Laurent, Jody E. Phelan, Leonid Chindelevitch, Timothy M. Walker, Daniela M. Cirillo, Anita Suresh, Timothy C. Rodwell, Paolo Miotto, Claudio U. Köser

**Affiliations:** 1 FIND, Grand-Saconnex, Switzerland; 2 Faculty of Infectious and Tropical Diseases, London School of Hygiene and Tropical Medicine, London, United Kingdom; 3 MRC Center for Global Infectious Disease Analysis, School of Public Health, Imperial College London, London, United Kingdom; 4 Oxford University Clinical Research Unit, Ho Chi Minh City, Vietnam; 5 Nuffield Department of Medicine, Center for Tropical Medicine and Global Health, University of Oxford, Oxford, United Kingdom; 6 Emerging Bacterial Pathogens Unit, IRCCS San Raffaele Scientific Institute, Milan, Italy; 7 Division of Pulmonary, Critical Care and Sleep Medicine, University of California, San Diego, California, USA; 8 Department of Genetics, University of Cambridge, Cambridge, United Kingdom; Brown University, Providence, Rhode Island, USA

**Keywords:** *Mycobacterium tuberculosis*, genotypic antimicrobial susceptibility testing

## LETTER

A limitation of the World Health Organization (WHO) mutation catalog, a global reference for genotypic antimicrobial susceptibility testing for *Mycobacterium tuberculosis* complex, is that it was derived and tested using the same data set, which may result in overfitting (1–4). Therefore, we welcome the effort by He *et al.* to assess the performance of both version 1 (V1) and version 2 (V2) catalog using an independent data set from China (5). However, we have concerns about how this was carried out.

The performance of sequencing for genotypic antimicrobial susceptibility testing depends on variant calling as well as the subsequent interpretation. Online tools, such as TB Profiler, combine both steps. By contrast, it is not clear based on the methods section how He et al*.* called variants prior to applying the WHO catalogs, which precludes replicating this step. This is important as numerous false-susceptible results appear to be due to missed variants rather than limitations of the two catalogs. For example, according to Table S2 of He et al. (5), sample 16-763 does not harbor *rpoB* Ser450Gly, and *eis* G-10A is not present in sample 14-38 using their pipeline. However, these mutations are present and should have been interpreted as resistance mutations for rifampicin and kanamycin, respectively, using both versions of the catalog, as shown in Table S1 of this letter.

Moreover, it is critically important to appreciate that WHO endorsed a set of additional grading rules that apply to existing and novel mutations (4). Specifically, any non-silent variants in the resistance-determining region of *rpoB* (codons 426–452) were assumed to confer rifampicin resistance (6). In addition, loss-of-function mutations (i.e., full-gene deletions, frameshifts, mutations that abolish the start codon, and premature stop codons) in some non-essential genes were assumed to confer resistance. For instance, *pncA* Ala134fs from sample 16-2174 from He et al. is not listed in the V2 mutation catalog report but is still subject to this rule and must be interpreted as resistant to pyrazinamide (4). Epistasis must also be considered when interpreting some mutations (e.g., *eis* promoter mutations cannot confer resistance when genetically linked with loss-of-function variants in the *eis* coding region [4, 7]). We acknowledge that these points should have been highlighted more clearly in the V1 report (2). Indeed, at least one other external assessment of the catalog initially overlooked these rules (1, 2, 8). To minimize oversight of these rules, we included section 2.1 in the V2 report (4) and are currently exploring new options to emphasize these parts of the catalog (e.g., synthetic FASTQ files that cover each rule [9]).

We encourage colleagues to independently validate the catalog and to share suggestions for improvements but urge them to pay attention to variant calling and to implement the catalog as detailed in the WHO report when doing so. For reference, Table 1 shows the expected performance of TB Profiler (v6.3.0) using its strict implementation of the V2 catalog (database name: who) (10). The full results are in Table S1.

**TABLE 1:** Performance of the second version of the WHO mutation catalog using TB Profiler^*a*^

Drug	True positives	True negatives	False positives	False negatives	Sensitivity (95% CI)	Specificity (95% CI)	Positive predictive value (95% CI)
Rifampicin	99	10	0	1	99% (94–99)	100% (72–100)	100% (96–100)
Isoniazid	90	9	0	11	89% (81–93)	100% (70–100)	100% (95–100)
Ethambutol	55	37	15	3	94% (85–98)	71% (57–81)	78% (67-86)
Pyrazinamide	53	43	2	12	81% (70–89)	95% (85–98)	96% (87–98)
Levofloxacin	62	43	2	3	95% (87–98)	95% (85–98)	96% (89–99)
Moxifloxacin	37	45	27	1	97% (86–99)	62% (50–72)	57% (45–69)
Amikacin	10	99	0	1	90% (62–98)	100% (96–100)	100% (72–100)
Streptomycin	64	41	0	5	92% (84–96)	100% (91–100)	100% (94–100)
Prothionamide	20	67	20	3	86% (67–95)	77% (67–84)	50% (35–64)
Capreomycin	8	99	2	1	88% (56–98)	98% (93–99)	80% (49–94)
Kanamycin	10	98	1	1	90% (62–98)	98% (94–99)	90% (62–98)

^*a*^ CI = Wilson score confidence interval.

## EDITOR'S NOTE

**Ed. Note**: The authors of the original article did not respond at the time of publication.

## SUPPLEMENTAL MATERIAL

Table S1 spectrum.02157-24-s0001.xlsx Detailed performance of the second version of the WHO mutation catalog using TB Profiler.

ASM does not own the copyrights to Supplemental Material that may be linked to, or accessed through, an article. The authors have granted ASM a non-exclusive, world-wide license to publish the Supplemental Material files. Please contact the corresponding author directly for reuse.
